# Evolution of informed consent in research: From the Hippocratic Oath to the tailored consent

**DOI:** 10.12688/openreseurope.17311.1

**Published:** 2024-04-17

**Authors:** Jaime Fons-Martinez, Carlos Murciano-Gamborino, Javier Diez-Domingo

**Affiliations:** 1 Vaccine Research Area, Foundation for the Promotion of Health and Biomedical Research of Valencia Region, FISABIO, Valencia, Spain; 2 CIBER de Epidemiología y Salud Pública, Instituto de Salud Carlos III, Madrid, Spain; 3 Universidad Católica de Valencia San Vicente Mártir. Facultad de Medicina y Ciencias de la Salud, Valencia, Spain

**Keywords:** Informed consent, research ethics, The Nuremberg Code, Declaration of Helsinki, Belmont Report, i-CONSENT, eConsent

## Abstract

**Background:**

Informed consent (IC) is essential in defending the autonomy of potential participants in clinical research. Despite the advances in research ethics, particularly in IC, the different guidelines and codes have not been fully implemented. Several studies have presented consent deficiencies that have resulted in unethical practices or poor understanding of the IC.

**Main body:**

This article reviews the evolution of IC, from its philosophical origins and initial use in the Ottoman Empire (16th century) to its use in clinical research today. It also presents the vision of the European project i-CONSENT (Grant Agreement number: 741856), whose main purpose is to improve the understanding of ICs in research and identifies the key components of a new paradigm to develop patient-centred ICs.

**Conclusions:**

In many cases, the IC has served to protect the investigator or sponsor from complaints. Different ethical guidelines have sought to make the IC a more useful tool, with little success. Today’s IC is mainly a bureaucratic and legal process that fails to consider the patient’s point of view. In this context, the Guidelines for Tailoring the Informed Consent Process in Clinical Studies provide alternatives to the current IC process, focusing on the patient’s opinions and making them part of the process, thereby improving clinical research quality.

## Background

The informed consent (IC) process is an essential procedure that ensures the fundamental rights and freedoms of the research subject, allowing them to voluntarily decide whether to participate in a study, and to withdraw without consequences at any time.

The changes in recent decades have consolidated the requirements for IC in research, making it an essential document to comply with ethical and legal standards. This has led to a highly regulated process, albeit with several gaps, as the different guidelines and codes have had limited success in practice.

These documents are often prepared solely by one of the stakeholders (usually the sponsor) without including other perspectives or interests. As a result, consent documents are long and complex, and are seen more as a bureaucratic act (a legal requirement and a 'contract' between the sponsor, the researcher, and the participant) than as an informational document. These complex documents mean that, in many cases, potential participants decide on their participation in a research study based exclusively on the oral information provided by the researcher, without even reading the consent form. This violates ethical standards: first, because the written information provided is difficult to understand by the patient; and second, the oral information provided by the researcher is beyond the scrutiny of the Ethics Committees or Good Clinical Practice inspections, contradicting efforts to trace all information within clinical trials. Oral information provided for consent represents the only gap in this traceability ^1^.

In the systematic review and meta-analysis conducted by Tam, Huy, Thoa *et al.* ^2^, no significant advances were found in the understanding of any element of IC over recent decades.

## Origin of informed consent

The idea of IC had its origin in Modernity, specifically in the political philosophy of the 16th century. It is built on the idea that human relationships cannot be based on a vertical relationship, in which one party has all the power and the other passively obeys. This idea, which eventually reached medicine four centuries later (20th century), represented a paradigm shift. It went from paternalistic Hippocratic medicine, where the doctor held all the knowledge (and made the decisions) and the patient was unable to decide for himself (and passively obeyed), to a relationship where the principle of autonomy governs; the patient makes the decisions after being informed by the doctor, making his status as a citizen prevail, with the right to be informed and make decisions about his health ^3, 4^. In the case of medicine, this shift has been slow and, in many cases, has had to be driven through legislative impositions or judicial decisions, leading in some cases to defensive practices ^3, 4^. Leclercq *et al.* ^5^ and Murray ^6^ compiled and analysed several relevant judgments regarding IC in the field of surgery, its procedures, or the information that it must include. Despite early use of the concept of IC in the judicial realm, the term “informed consent” was only introduced into the judicial lexicon in 1957, when an appellate judge in California issued his written opinion in the case of Salgo *vs.* Leland Stanford Junior University Board of Trustees ^7^.

In other cases, members of the scientific and/or medical society itself have demanded changes. In 1902, the psychiatrist Albert Moll emphasized the need for IC after collecting 600 cases of unethical non-therapeutic research on humans ^8^. The physiologist Walter Cannon, in 1916, proposed to the American Medical Association (AMA) that they mandate IC for research. However, the AMA rejected his proposal, arguing that they considered that misconduct was not a problem of the research itself, but of dishonest researchers, and believed that better research and clinical care would come through trust, and not through regulation ^9^. Basing the consent on a relationship of trust precludes traceability of the whole process, especially the oral explanation of the investigator. Despite this paradigm shift and the fact that IC focuses on autonomy, its ethical foundation requires correct articulation of the four classic principles of modern bioethics: non-maleficence, justice, beneficence, and autonomy. As Simón indicates: “ *what ‘must be done’, therefore, will be the result of the prudent weighing of the obligations derived from respect for autonomy and beneficence, in a framework pre-established by non-maleficence and justice”* ^3^.

As we have already mentioned they are two different fields where the IC is used in medicine: treatment and research. Even both of them are founded on the principle of autonomy, consent for treatment is mainly based on case law while consent for research is based on ethical codes, statutes and administrative regulations ^10^.

## First informed consent documents

The use of consent documents (in the form of contracts and with obvious differences with current consents) dates back to the 16th century, in the former Ottoman Empire, where contracts were signed before carrying out surgical interventions. In this regard, Sayligil and Ozden ^11^ present a private consent form registered in Sharia Court Records (Ser’iyye Sicilleri) found in Bursa, dated 1524; Selek ^12^ identifies a contract from 1539, signed in Gaziantep, Turkey (formerly known as Aintab); and Ajlouni ^13^ shows a 1677 contract signed in the Ottoman Tripoli (now Libyan territory). These documents identify the contractor (patient or his/her father), the doctor, the court before which the contract is presented, the patient's illness, the procedure (including in some cases postoperative treatment), fees, the absence of responsibility of the doctor in the event the patient is harmed or dies, and are signed by the patient (or his/her father) and the doctor in the presence of witnesses (identifying them).

In the Western world, the first mentions of IC date back to the 18th century, also in the field of surgery. The orthopaedists Baker and Stapleton, without informing or obtaining prior approval from the patient (Slater), refractured and used an experimental medical device to align and straighten his non-union fractured leg, which was being treated with bandages. In the judgment of the trial “ *Slater vs Baker and Stapleton*” (1767), the court ruled in favour of the patient, saying that a patient should be informed of the procedure, so that he/she takes courage and charge of the situation, and can face the operation ^14^.

Altschuler ^15^ notes that one of the first descriptions of the concept of IC appears in Edgar Allen Poe's novel " *The facts of the case of M. Valdemar*" (1845), in which the protagonist asks Mr. Valdemar for permission to practice a hypnosis experiment on him before his death.

Suárez-Obando and Ordoñez ^16^ and Vollmann and Winau ^8^ identify the First and Second Prussian Directives on Research as the predecessors of today’s IC in research. The First Prussian Directives on Research (1891) regulated research with prisoners, and included, as a requirement, the consent of the prisoner to be treated with the experimental treatment. The Second Prussian Directive on Research (1900) indicated that in medical interventions that did not have the purpose of diagnosis, treatment or immunization, neither minors nor the mentally incompetent could participate. In addition, it indicated that the subject must give his/her “unambiguous consent” after a “proper explanation of the possible negative consequences" of the intervention. This directive also indicates that the medical doctor is the only one who can perform or authorize research interventions, and that the fulfilment of all requirements and other circumstances of the case had to be documented in the medical record ^8^.

 Suárez-Obando and Ordoñez also highlight the pioneering role of the Reed Commission in its investigations on yellow fever in Cuba (1900). It included a document similar to a contract that explained the risks of participating in the study, the lack of an effective treatment against the disease, the will of the subject to participate in the research, and the conditions of participation ^16^.

## Informed consent in research after the Second World War: From the Nuremberg Code to the present day

### The Nuremberg Code (1947)

Even though the Nuremberg Code has never been officially adopted as law by any nation or as ethical guidelines by any major medical association, due to its profound influence on global human rights law and medical ethics, some authors identify it as the most important document in the history of medical research ethics ^17^.

During what is commonly known as the Nuremberg Trials, the International Military Tribunal prosecuted the leading Nazi authorities, the Trial of the Major War Criminals being the best known. A second group of trials of minor war criminals, under Control Council law no. 10 in the Military Courts of Nuremberg of the United States, included the so-called “Doctors' Trial”. Twenty of the 23 defendants were physicians, accused of torture and murder in the conduct of medical experiments on concentration camp inmates. These physicians were charged with war crimes and crimes against humanity; they were accused of having violated the Hippocratic Oath and having behaved in a way incompatible with their profession. The Nuremberg Code (1947) grew out of this trial, being drafted by the International Tribunal of Nuremberg and advised by Leo Alexander and Andrew Ivy, the American doctors who participated in it. The participation of Alexander and Ivy was fundamental. Before and during the trial, these doctors provided the chief prosecutor, Telford Taylor, with different documents with ethical, legal and scientific principles and requirements for human research, which already included free and informed consent. The memorandum entitled “ *Ethical and Non-Ethical Experimentation on Human Beings*” and the document “ *Principles of Ethics Concerning Experimentation with Human Beings*,” adopted by the AMA House of Delegates in December 1946, were among these documents. The Nuremberg Code identified the 10 basic principles that medical experiments should meet to satisfy moral, ethical and legal concepts. These included the specification that researchers must always recruit competent research subjects who understand the nature of the research and who voluntarily consent to participate. For this reason, the subjects must have sufficient knowledge and understanding to be able to make a conscious decision, which derives from a prior explanation (principle 1) and the subject’s right to withdraw their participation from the research (principle 9) ^8, 9, 17, 18^.

While some American researchers understood the need for these principles (although they sought to moderate the strict language of the Code), many others considered that the Nuremberg Code did not apply to them, as it had no legal authority and they considered it a response to the experimental work done by Nazi researchers ^9^. As De Abajo points out, doctors never felt any connection to the Code, which was largely alien to the profession, drawn up "only" to judge criminals.

It should be noted that Ivy acknowledged during the Trial that there was no specific code for medical research prior to 1946, and that the principles adopted by the AMA were expressly formulated for the Doctors’ Trial ^17^. On this point, Ghooi criticizes the Nuremberg Code’s lack of originality, and points out that six of its 10 principles (including the need for IC) derive directly from the Guidelines for Human Experimentation issued during the Weimar Republic by the German minister of the Interior in 1931, with no mention of them in the Code ^18^.

### Declaration of Helsinki (1964-last revision 2013)

In June 1964, the World Medical Association (WMA) adopted the Declaration of Helsinki, which has been amended nine times to date, the last time in October 2013.

The Declaration of Helsinki was based on the Nuremberg Code and mainly on the document “ *Principles for those in Research and Experimentation*” (WMA, 1954) ^19^. It was developed “ *as a statement of ethical principles for medical research involving human subjects, including research on identifiable human material and data. The Declaration is intended to be read as a whole and each of its constituent paragraphs should be applied with consideration of all other relevant paragraphs*.” ^20^

This Declaration gives great importance to IC, a term that appears to have been employed for the first time in a medical research ethics document in the first revision of the Declaration, adopted in Tokyo (1975). The current version (2013) contains a section with eight articles, from 25 to 32, dedicated exclusively to IC. This section includes, among other aspects: 1) the need for IC and willingness to participate; 2) that potential participants must receive adequate information, including the right to refuse to participate in the study or to withdraw consent to participate at any time without reprisal; 3) the importance of ensuring that the potential subject has understood the information; 4) the need for consent by a legally authorized representative when the person is incapable of giving informed consent; 5) cases in which the individual should not be included in the research; 6) circumstances when consent does not need to be obtained; 7) the importance of avoiding undue influence; and 8) how to proceed with identifiable human material or data.

Another important contribution of the Declaration of Helsinki is the introduction of the need for an additional external guarantee, provided by the supervision of an independent committee, which is responsible for examining the study protocol, providing comments and suggestions to the investigator, and approving it, before the study begins. This figure was introduced in the second version of the Declaration, in Tokyo (1975).

### Belmont Report (1979)

Despite the advances in research ethics achieved by the Nuremberg Code and the Declaration of Helsinki, some investigators and studies continued disregarding the basic ethical standards, such as IC. In 1966, Henry Beecher published the article " *Ethics and Clinical Research*", highlighting the lack of ethics of many research studies published in scientific journals, and emphasized the importance of obtaining IC, which he identifies as the most important component of ethics in research. He also advocated the non-publication of results obtained from unethically obtained data ^21^.

In 1972, Jean Heller published an article in the *Washington Star* and the *New York Times*, in which she denounced a study sponsored by the US Public Health Service on the effect of untreated syphilis in poor rural African-American subjects. The study was entitled *“USPHS Untreated Syphilis Study at Tuskegee”*. The study that began in 1932, when the treatment was ineffective and quite toxic, lasted 40 years (although its initial expected duration was six months). Participation in the study provided participants with free medical examinations, free meals, and burial insurance, but not the benefit of providing IC. The participants were not informed about medical advances in the treatment of syphilis, despite the fact that the efficacy of penicillin treatments was demonstrated in 1945. They were kept without treatment with terrible consequences for their health ^22^.

The impact of both articles led to legislative changes in the United States and the creation in 1974 of the National Commission for the Protection of Human Subjects of Biomedical and Behavioral Research. This Commission published the Belmont Report in 1979 ^23^. The Report summarizes the basic ethical principles identified by the Commission during the 4-day discussion that took place in February 1976 at the Smithsonian Institution's Belmont Conference Center, and during the Commission's monthly deliberations held over a period of nearly four years. The Report is a statement of basic ethical principles and guidelines on research involving human subjects ^23^.

The Belmont Report marks a before and after for IC. It is divided into three parts:

- Boundaries Between Practice and Research: distinguishes the practice of an accepted therapy from biomedical and behavioural research. While practice is aimed at enhancing the well-being of an individual patient, research seeks to test a hypothesis, draw conclusions, and obtain generalizable knowledge.
- Basic Ethical Principles: defines the three basic ethical principles: Respect for Persons, Beneficence, and Justice. Respect for Persons, which is the one that interests us most when talking about IC, suggests that individuals should be treated as autonomous entities and that those with diminished autonomy should be protected, and indicates that, *“in most cases of research involving human subjects, respect for persons demands that subjects enter into the research voluntarily and with adequate information”.*
- Applications: identifies the requirements involved in applying the general principles, which are IC, Assessment of Risks and Benefits, and Selection of Subjects.

According to the Report, IC is based on the triad **Information, Comprehension and Voluntariness**:

- **Information**: several specific codes contain recommendations to ensure that the subject has sufficient information, but *Information* still has several issues in terms of the criteria to judge the amount and sort of information that should be provided. However, problems may arise, when giving information on a relevant issue is likely to impair the validity of the research.
- **Comprehension**: a person's ability to understand is a function that depends on intelligence, rationality, maturity, and language. The information must be adapted to the capabilities of the subject. Moreover, the manner and context in which the information is presented is as important as the information itself.

Unlike the Declaration of Helsinki, which indicates that the participation of subjects who cannot give their consent must be authorized by their legal representative, the Belmont Report indicates that participation must be authorized by a third party. These parties should be those *“most likely to understand the incompetent subject's situation and to act in that person's best interest”.* Subjects with limited comprehension should be informed and their objections should be honored, except when the research provides them with a therapy otherwise unavailable.

Investigators are responsible for ensuring that the subject has understood the information. A verbal or written comprehension test may be useful in this respect.

- **Voluntariness**: valid consent must be given voluntarily, free of coercion (threat of harm is presented intentionally to obtain compliance), undue influence (offering an excessive, unwarranted, inappropriate or improper reward or other overture for participating) or unjustified pressure (when people in a position of authority or with influence urge the subject to participate).

Although supervision of the principle of autonomy by independent committees is not required in the Report, it recognizes the important role of these committees in assessing beneficence and any potential risks and benefits associated with the research ^1^.

### ICH Guidelines for Good Clinical Practice (1996 – last revision 2017)

The International Council for Harmonisation of Technical Requirements for Pharmaceuticals for Human Use (ICH) was created in 1990 with the mission of achieving *“greater harmonisation worldwide to ensure that safe, effective, and high quality medicines are developed and registered in the most resource-efficient manner”* ^24^. This harmonisation is achieved through the development of the ICH Guidelines.

Frías ^25^ indicates that these standards were driven by the desire to combine criteria and that they represent a " *set of obligations of sponsors, monitors and researchers who participate in conducting clinical trials*".

Guideline E6 of the ICH Guidelines refers to Good Clinical Practice (GCP). The first version was published in 1996, and was used from 1997 to 2017, when it was replaced by Revision 2 (R2). A new revision of the guideline (R3) began at the end of 2019 - early 2020.

Since its first version, the Guidelines have dedicated section 4.8 to *“Informed Consent of Trial Subjects”.* This section includes relevant information, such as the obligation to have a favourable opinion by the Institutional Review Board or Independent Ethics Committee; aspects of autonomy and voluntariness (this includes aspects such as the need for consent, the importance of informing, the non-acceptance of undue coercion or influence, or the importance of using a language understandable to the potential participant or their legally accepteable representative *.*); aspects related to IC when the research includes minors, people unable to provide consent or in emergency situations; minimum information that must be given to the subject or the need to use simple and practical language, among others. These guidelines also give importance to oral information in the relationship between the research team and the subject, and it is considered that both oral and written information must be understandable.

### CIOMS Guidelines (1982- last version 2016)

The Council for International Organizations of Medical Sciences (CIOMS) was founded in 1949 under the auspices of the World Health Organization (WHO). In 1982, CIOMS and the United Nations Educational, Scientific and Cultural Organization (UNESCO), in association with the WHO, drew up the “ *Proposed International Ethical Guidelines for Biomedical Research Involving Human Subjects”*, with the aim of providing “ *internationally vetted ethical principles and detailed commentary on how universal ethical principles should be applied, with particular attention to conducting research in low-resource settings”.*

The fourth version of the guidelines (2016) ^26^ establishes 25 guidelines and four appendices, with an introduction and a brief description of previous instruments and guidelines. These guidelines are a very valuable text in research ethics, especially with regard to IC, since several of them address related aspects that are of great interest. For example, guideline 7, “ *Community engagement”*, incorporates the idea of including potential participants and communities in the design of the IC process. In addition, it dedicates Guideline 9, “ *Individuals capable of giving informed consent*”, exclusively to IC and Guideline 10 to the “ *Modifications and waivers of informed consent*”. The IC dilemma in biobanks and in future research is addressed in Guidelines 11, “ *Collection, storage and use of biological materials and related data*”, and 12, “ *Collection, storage and use of data in health-related research*”. Guidelines 15 to 21 include aspects related to consent in specific groups and situations, such as research with vulnerable persons and groups, adults incapable of giving IC, children and adolescents, or women (with particular attention to research during pregnancy and breastfeeding). Research in disaster and disease outbreaks are also included in these guideline situations, as are cluster randomized trials. CIOMS guidelines should be taken into account when discussing research ethics, and especially IC.

## What are the main challenges of informed consent in research nowadays?

Despite major advances in research ethics and the increasing regulation of informed consent, there are a number of challenges that have persisted over the past 50 years and are difficult to overcome ^27^. These include:

### Length and difficulty of comprehension

In an attempt to increase transparency and ensure that the potential participant has the necessary information to make an informed decision on whether or not to participate in research, informed consent has become increasingly regulated and standardised, resulting in lengthy documents ^28^. This increase in the content of informed consent forms does not guarantee that they contain all important information for potential participants or that they properly understand it, in fact, too much information can impair understanding ^29^.

In addition, even the use of plain language has been widely recommended, most consent materials still include complex language ^30^. This is an important issue, as health information materials (including informed consent) are often too complex for a significant proportion of the population who lack the necessary skills and health literacy levels to adequately understand them ^31^.

### Focus

Often, both the sponsor and the researchers have considered informed consent primarily as a bureaucratic act and a legal requirement, centred on the document and the act of signing.

The informed consent documents have generally consisted of lengthy texts that are difficult to understand, written in complex and technical language ^2, 32^. Moreover, in most cases, they are written by the sponsor, without taking into account the perspectives of other stakeholders, and are sometimes constructed more as defensive documents designed to serve the interests of institutions and sponsors than for informational purposes ^27^. Documents should be understood as the basis of a meaningful exchange between researcher and subject rather than as an end ^33^.

Rather than focusing on the signature, informed consent should be understood as a process rather than a punctual act ^34^. Indeed, the emphasis on the signature and the idea that it entails the participant's adequate understanding of the information is increasingly being questioned ^35^.

### Respecting diversity of values and backgrounds

The inclusion of people with different characteristics and backgrounds in research is extremely important, to the extent that some authors consider that improving diversity in clinical trials is a moral and scientific imperative ^36^. Informed consent forms often adopt standardised approaches that do not take into account the characteristics and are not tailored to the needs, preferences and interests of potential participants. Nevertheless, the characteristics of the population must be taken into account when designing informed consents, for example, whether the person is illiterate or the culture to which he or she belongs. It is important to bear in mind that norms vary across cultures ^37^ and these may affect informed consent and concepts such as autonomy, voluntariness, altruism or even illness.

### Technological advances

Research methodologies and ways of communicating have changed over the years. These advances have also brought new challenges and opportunities for informed consent, enabling new approaches to both how informed consent is presented and how it is obtained. Grady *et al.* ^27^ identify several challenges and areas of research in the use of electronic and digital informed consent, in terms of disclosure, comprehension, voluntariness and authorisation.

The systematic review done by Gesualdo *et al.* on the use of digital tools in the informed consent process ^38^ highlights their potential positive impact on improving participants' understanding and satisfaction, and indicates that at least it seems clear that they do not affect them negatively. The authors also highlight the lack of standardised methods for assessing the impact of digitally supported informed consent processes.

The use of social media to recruit participants for clinical trials is increasingly common, but it also brings with it ethical challenges for which there is no clear, agreed-upon answer. Social media recruitment requires legal and ethical standards to be applied in a context that may be unfamiliar to researchers and ethics committees ^39^.

Caplan and Friesen ^40^ explain the potential benefits of using social media in recruiting participants for clinical trials. They highlight the control it allows and its advantages to reach more diverse populations, by being able to deliver different messages to different audiences or to select the profile of the person who will receive them, based on age, location, language, education, marital status or occupation. 

### Investigator’s communication skills

Several authors have highlighted the importance of communication between the physician and the patient/participant in creating a favourable and trusting environment for the discussion of different health issues. This contributes to patient satisfaction. In many cases physicians overestimate their communication skills ^41^.

During the informed consent process, the conversation between the investigator and the potential participant is key. In this regards, Hayman, Taylor, Peart *et al.* ^42^ identified researcher’s verbal explanation as the most useful information for the parents who were invited to enrol their infant in a research project, over written information; Nishimura, Carey, Erwin *et al.* ^43^ included the extended conversation between researcher and participant as one of the elements to improve comprehension of the IC; Stevens and Pletsch ^30^ indicate that participants trust health professionals more than what is written in the IC and give much weight in their decision to participate in the study to the assessment of the health professional recommending the study, rather than weighing the information received itself; and Tam *et al.* ^2^ identify the explanatory ability of researchers as a factor influencing participant understanding.

### Biobanks

There are different types of informed consent applicable to biobanks: specific/close consent; broad consent; blanket/open consent; dynamic consent. The use of one type of consent or another has different implications for the donor's autonomy and control over the use of the samples he/she has donated in different research studies. Sometimes these differences are not clear to the donor or even to some researchers who include the collection of biological samples in their studies.

### Pandemics

The COVID-19 situation we have been experiencing since the first quarter of 2020 has highlighted the importance of having guidelines on how to act in health pandemic situations from all fields, including medical research and informed consent.

The global COVID-19 pandemic created a new scenario in medical research, both in terms of how to combine efforts and share information internationally and how to deal with legal and ethical dilemmas, including those related to informed consent. In Europe, the European Medicines Agency (EMA), as well as national medicines agencies, responded to this new challenge by issuing guidances on the management of clinical trials during the pandemic. These guidances included proposals to make clinical trials more flexible during this period so that they could continue. Sixteen agencies (15 national agencies and the EMA) have included guidance on obtaining the IC, in order to prevent the spread of the virus and to comply with restrictions imposed by governments during the pandemic. These guidances included alternative forms of consent or even exceptions to consent to avoid overcrowding of health care facilities and the need for participants to travel, and proposed alternatives to the traditional paper-based IC forms ^44^.

## i-CONSENT (2017–2021)

The European Commission echoed most of these difficulties and challenges, and in October 2015 published a call for proposals (reference number SwafS-17-2016) under the title “The Ethics of informed consent in novel treatment including a gender perspective”, which was resolved in favour of the project entitled “Improving the guidelines of informed consent, including vulnerable populations, under a gender perspective (i-CONSENT)” (Grant Agreement 741856).

This four years project (2017–2021) aims to improve the informed consent processes in clinical research, making them easier to understand and tailored to the potential participant’s needs and preferences. The final product of the project are a series of guidelines to improve Informed Consent process, including vulnerable populations, under a gender perspective and relying on ICT tools ^45^.

These guidelines, entitled “Guidelines for Tailoring the Informed Consent Process in Clinical Studies”, have been written in accordance with other existing guidelines and legal documents, and should be read in conjunction with them ^46^. They aim to “ *help researchers utilise bidirectional and continuous communication during the process of informed consent, without losing sight of vulnerable populations, multiculturalism and gender perspectives*” ^1^.

i-CONSENT defines the IC process as a *“bidirectional communication process that begins with the first contact with the potential participant and continues throughout the study until its end. During this process, continuous feedback and communication between the participant and the research team are essential”* ^46^.

Furthermore, the Guidelines for Tailoring the Informed Consent Process in Clinical Studies aim to help improve IC in the future, for which they propose a paradigm shift in which the research participant is central to the IC process. i-CONSENT envisions an IC that:

- Is considered a process that adds value, rather than a bureaucratic procedure or legal requirement.
- Ceases to be a defensive document and focuses on the participant.
- Does not focus on the signature and is seen as a process that extends from the first contact with the potential participant to the end of his/her participation in the study.
- Takes into account the point of view of the most involved stakeholders, such as potential participants and investigators, and not just the sponsor.
- Incorporates potential participants in the process of preparing informative materials from the beginning (design, co-creation, revision) through participatory methodologies, such as design thinking or other qualitative or quantitative methodologies to determine their opinion, such as surveys, interviews or focus groups.
- Improve participants' health literacy throughout the consent process.
- Is inclusive and tailored to the participant.

The methodology employed to develop the guidelines used a multidisciplinary approach. Several reviews of legal, ethical and scientific documents have been conducted on different aspects of IC. Qualitative techniques (such as focus groups, nominal groups, and design thinking) have been used with potential participants, representatives of patient associations, investigators, representatives of the pharmaceutical industry, ethicists, members of ethics committees and regulators to obtain information and explore their points of view, needs and interests. Social media analysis has also been carried out.

Following the recommendations included in the guidelines, IC materials were prepared for three study cases (mock scenarios/studies) ^47^. These materials were tested in three different populations and countries with very positive results. Additionally, a panel of experts assessed the appropriateness of some of the recommendations included in the guidelines ^48^.

The recommendations included in the guidelines have been already used in a real clinical trial: VIGIRA ^49^.

## The pillars of informed consent adapted to the participant

It is crucial that IC enables a person to:

- Make an informed and autonomous decision about their participation in a study.
- Re-evaluate their participation throughout the study, and understand their freedom to withdraw at any time.

The acronym i-CONSENT contains the core elements of a comprehensive consent process ( Table 1).

**Table 1.** The i-CONSENT acronym and core elements of the consent process.

I	Information	Complete and clear information is essential for the potential participant to be able to make an autonomous decision.
C	Co-creation	The inclusion of potential participants during the design and review of study information materials is key to ensure that they are understandable and address the target population’s needs and preferences.
O	Ongoing process	Consent should be a two-way continuous communication process that begins at first contact with the potential participant, and continues until the end of the study.
N	New technologies, methods, and processes	The consent process should include technical and methodological innovations to facilitate the participant’s experience. Their appropriateness from a social, methodological, legal and ethical point of view should always be taken into consideration.
S	Self-determination (autonomy)	Autonomy is a fundamental principle. The purpose of the informed consent is to ensure that the potential participant is able to make an autonomous and free decision.
E	Empowerment	Participants should be empowered to make their own decisions.
N	Nonstandard (inclusive and tailored)	Research should be inclusive to meet the needs of the potential participants and respect the basic bioethics principle of justice. There is no single best way to conduct the consent process. The ‘ideal’ solution will differ in every setting and therefore needs careful design. Where possible, the consent process should be tailored to the needs of the target population.
T	Trusted	Good practices are essential to build trust between investigators and potential participants, and to increase society’s trust in research.

Source: Guidelines for Tailoring the Informed Consent Process in Clinical Studies ^46^.

The Guidelines for Tailoring the Informed Consent Process in Clinical Studies recommend that the IC process incorporate key interventions designed to enhance autonomy and inclusion. As noted in the expert assessment of the recommendations included in the guidelines, the four keys to improving understanding of IC in clinical trials are: *“(1) consider consent a two-way continuous interaction that begins at the first contact with the potential participant and continues until the end of the study; (2) improve investigators’ communication skills; (3) co-create the information; and (4) use a layered approach, including information to compensate for the potential participant’s possible lack of health literacy and a glossary of terms*” ^48^.

These key recommendations, together with those contained in the rest of the guidelines, have the potential to result in higher quality, lower cost and ethically justified research. The potential impact of better information and communication is high at the ethical level (safeguarding people's autonomy) and in research in general.

## Conclusions

The principle of autonomy has reached the field of medicine later than other disciplines, due to the strong Hippocratic tradition and because of the sensitivity of health (individual and collective) itself. From the 16th century to the present day, consent in many cases has had a defensive purpose (to safeguard the doctor or sponsor from possible complaints) rather than an informative one, becoming a perversion of the consent itself. Legislation and court rulings have been placing emphasis on the need for consent, and the contents that it must include have been expanded.

The different ethical guidelines that have emerged have sought to make consent a more useful tool and closer to its objective (to defend the autonomy of the participant), but their non-mandatory nature has meant they have less practical impact.

The problems presented by IC today — such as to be focused on the signature; considered mainly a bureaucratic act and a legal requirement by the sponsor and research staff; their excessive length; use of very complicated language and medical jargon; being drawn up by only one of the interested parties, usually the sponsor, without taking other points of view into account; or that a large part of the potential participants deciding whether or not to participate based on the discussion with the researcher, without even reading the informational materials — drastically diminish their success as a guarantor of the participant's autonomy.

Despite the availability of several guidelines, sponsors and researchers do not use them properly. Thus, involving all stakeholders, including regulators, is key to moving from theorizing to practice.

The Guidelines for Tailoring the Informed Consent Process in Clinical Studies, prepared within the framework of the i-CONSENT project, promote a change in the way of conceptualizing IC. Thus, it becomes a process that provides added value and prevents it from becoming a bureaucratic act focused on the participant’s signature on the IC form, having as a central concept the implementation of a participant-centred design and the improvement of effective communication.

The guidelines provide high level recommendations to improve the IC process from the drafting of consent materials until the end of the study. They also include a step-by-step description of the IC process and a checklist to implement comprehensive and inclusive IC, as well as 14 fact sheets and six tools with recommendations and examples to put ethical considerations into practice. 

## Ethics and consent

Ethical approval and consent were not required.

## Data Availability

No data are associated with this article.
