# Supplementary material for: Evolution of informed consent in research: From the Hippocratic Oath to the tailored consent
Source: Open Res Eur. 2024 Apr 17;4:72. [Version 1] doi: 10.12688/openreseurope.17311.1 (PMC11399764; doi:10.12688/openreseurope.17311.1)
Supplement: Peer review history

# Reviewer report: Reviewer response for version 1

**DOI:** 10.21956/openreseurope.18711.r40698

**Published:** 2024-09-13

**Authors:** Vicente Bellver Capella, Lukas Romero-Wenz

**Affiliations:** 1 Universidad de Valencia, València, Spain; 2 Legal and Political Philosophy, Universitat de Valencia, Valencia, Valencian Community, Spain

The document is directly related to a research project funded by the European Union that examined the challenges facing informed consent today and the proposals to ensure that informed consent documents are truly a guarantee of the fundamental rights of the subject of the investigation and not a bureaucratic procedure with a primarily defensive objective.

 The paper consists of three parts. The first presents a summary of the milestones in the history of informed consent, with some especially relevant information little known to the general public. The second refers to the main challenges that informed consent presents today. And, finally, and echoing the results of the i-Consent project, the principles that should inform the guidelines that are approved for the preparation of informed consent documents are presented.

Is the argument persuasive and supported by appropriate evidence?

Yes

Is the work clearly and cogently presented?

Yes

Is the topic of the essay discussed accurately in the context of the current literature?

Yes

Does the essay contribute to the cultural, historical, social understanding of the field?

Yes

Reviewer Expertise:

Bioethics and Biolaw

We confirm that we have read this submission and believe that we have an appropriate level of expertise to confirm that it is of an acceptable scientific standard.

# Reviewer report: Reviewer response for version 1

**DOI:** 10.21956/openreseurope.18711.r40697

**Published:** 2024-08-14

**Authors:** Julia Wade

**Affiliations:** 1 Bristol Medical School (PHS), University of Bristol, Bristol, England, UK

- Is the topic of the essay discussed accurately in the context of the current literature?

The authors refer to past and current literature in a way that is helpful to the reader in pulling relevant literature together.

 One useful paper that currently isn't referenced is Dixon Woods et al (2007). This paper argues that it is not possible to 'fix' information provision such that parallel discussion alongside written information becomes imperative.

 Great to see mention of layered consent; other work in this area could be referenced, e.g. Symons et al (2022).

- Is the work clearly and cogently presented?

The authors present a valid argument, namely that research informed consent processes meet the requirements of ethics committees but at the expense of providing information that is relevant and easily understood by patients.

- Is the argument persuasive and supported by appropriate evidence?

Yes, the argument is valid and is well supported by the literature referenced.

 It would be very helpful if the authors could clearly distinguish between the principle of informed consent vs the process of ensuring consent is fully informed vs different ways to operationalise the process of ensuring informed consent is given to take part in research.

 The principle is very much that consent should be voluntary and based on understanding what the research involves. History has shown us that we need guidelines to ensure this principle is followed where possible and reasonable alternatives are in place where not, and this is clearly and helpfully documented here. Where these guidelines have not been in place, researchers/healthcare professionals may abuse the power they have over patients/potential participants.

 How that is currently operationalised can be critiqued.  How do we best inform people and how do we best ensure that they understand to what they are consenting? There are 3 key principles underpinning informed consent 1) voluntariness; 2) understanding; 3) capacity. Voluntariness is probably of higher priority than the principle of understanding; writing as someone who has repeatedly consented to take part in research without reading all the participant facing information. Ideally we want people to be fully informed, but as a participant, I can waive my right to read the entire information sheet.

 Three sets of stakeholders have subtly different agendas as their top priority: potential participants have their own current interests as priority, but also frequently altruism to help others in future; Ethics committees set out to protect patients from harm during the research process; Researchers set out to recruit to their research in order to benefit patients in future and with this in mind, try to be transparent and clear in what the research involves.  Inevitable tensions arise from these different priorities: insistence by ethics committees on communicating potential risk of harm results in the current very long participant information sheets which function as a barrier to patient understanding and giving truly informed consent.

 In the abstract and introduction, it would be helpful if the authors could distinguish between the principle of informed consent, the range of processes by which to ensure informed consent is given and be clear to distinguish these from the participant information sheet/consent form (currently referred to as 'the document', second paragraph of p3).  Currently both the abstract and text refer to 'poor understanding of the IC' and I believe this refers to the informed consent form or participant information sheet (a 'document') rather than the whole process of ensuring information provision, understanding and voluntariness.

 In fact it is the IC **process** that should be made more patient-centred, not just the IC form and it was the IC process that was the focus of i-CONSENT. So helpful to be very clear in specifying whether a reference is being made to the process vs the IC form throughout to avoid confusion.

- Does the essay contribute to the cultural, historical, social understanding of the field?

The essay provides helpful historical perspective to account for processes currently adopted when seeking to ensure fully informed consent is given for participation in research.

 found it helpful to have this historical context as it accounts for many of the processes in place today, some of which, as the authors argue, actually function as a barrier to patient understanding of what they consenting.

 Thank you for the opportunity to review this essay, I found it informative and extremely helpful in mapping the historical and current context.

Is the argument persuasive and supported by appropriate evidence?

Partly

Is the work clearly and cogently presented?

Partly

Is the topic of the essay discussed accurately in the context of the current literature?

Partly

Does the essay contribute to the cultural, historical, social understanding of the field?

Yes

Reviewer Expertise:

Optimising informed consent within Trials research.

I confirm that I have read this submission and believe that I have an appropriate level of expertise to confirm that it is of an acceptable scientific standard, however I have significant reservations, as outlined above.

## References

[1] : Consumer perspectives on simplified, layered consent for a low risk, but complex pragmatic trial. *Trials* .2022;23(1) : 10.1186/s13063-022-07023-z 1055 10.1186/s13063-022-07023-z 36578070 PMC9795139

[2] : Beyond "misunderstanding": written information and decisions about taking part in a genetic epidemiology study. *Soc Sci Med* .2007;65(11) : 10.1016/j.socscimed.2007.08.010 2212-22 10.1016/j.socscimed.2007.08.010 17904716

# Reviewer report: Reviewer response for version 1

**DOI:** 10.21956/openreseurope.18711.r40693

**Published:** 2024-05-25

**Authors:** Dirk Lanzerath

**Affiliations:** 1 University of Bonn, Bonn, Germany

The article provides an excellent overview of the development of informed consent. It is particularly interesting that it is not only based on European developments and documents.  The authors take a critical look at the now excessive flood of information in informed consent and the question of whether the purely administrative process of obtaining a signature really fulfils the actual aim of freely and informed consent to participate in a research project. Digitalisation of processes and harmonisation of procedures are also addressed

 I would like to mention the following suggestions for revision or additions:

 - Individual consent is not common in all countries, but the group/tribe, partner or other persons play an important role. This should be noted and also changes questions about e-consent, for example.

 - The title is somewhat misleading because it is not about research but exclusively about medical research. Therefore, "medical" for research should be added to the title.  In psychology and the social sciences, for example, there are other problems and lines of development than in medicine.

Is the argument persuasive and supported by appropriate evidence?

Yes

Is the work clearly and cogently presented?

Yes

Is the topic of the essay discussed accurately in the context of the current literature?

Yes

Does the essay contribute to the cultural, historical, social understanding of the field?

Yes

Reviewer Expertise:

research ethics, bioethics

I confirm that I have read this submission and believe that I have an appropriate level of expertise to confirm that it is of an acceptable scientific standard.
